# Deadly injuries through recoilless anti-tank weapons while military shooting practice—two case studies from Germany and Switzerland

**DOI:** 10.1007/s00414-020-02301-4

**Published:** 2020-04-28

**Authors:** Katharina Jellinghaus, Charlotte Scherer, Edouard Stauffer, Petra Urban, Michael Bohnert, Beat P. Kneubuehl

**Affiliations:** 1 Institute of Forensic Medicine, Julius-Maximilians-University, Versbacher Str. 3, D-97078 Wuerzburg, Germany; 2 PROMED Laboratoire médical SA, Route Ancienne Papeterie 131, CH-1723 Marly, Switzerland; 3 Institute of Biological Anthropology, Medical Faculty, Albert-Ludwigs-University, Hebelstr. 29, D-97104 Freiburg, Germany; 4 Bpk Consultancy GmbH, Forstweg 25, CH-3603 Thun, Switzerland

**Keywords:** Anti-tank weapon, Military shooting, Davis gun, Anti-tank rocket, Ballistics

## Abstract

In this casuistry, two accidents from Germany and Switzerland are presented that happened during the shot of recoilless anti-tank weapons. In both cases, the injuries led to the death of two soldiers: A 22-year-old soldier in Germany was struck by the counter mass of a so-called Davis gun which had been fired by a comrade during a firing exercise; he died from his severe injuries, especially in the abdominal part of the body. As a peculiarity of the wound morphology, it was found to be a thick-layered, metallic, gray material in the wound cavity, which corresponded to the material of the counter mass that was ejected opposite to the shooting direction. The other case took place in Switzerland, where a 24-year-old soldier was seriously injured during an exercise with portable anti-tank rockets. At the time the shot was fired, he stood behind the launcher and was hit by the propulsion jet of the rocket motor. He died as well from his severe injuries, which were located at the chest done by the gas jet and by the very high pressure. In both cases, two different causes of death were present: massive blunt violence in the first case versus a jet of hot gases of very high speed and temperature in the second case.

## Introduction

The defense against armored vehicles (tanks, armored personnel carrier) requires grenades with a weight of several kilograms; due to the large muzzle momentum (mass times speed), it can only be fired from heavy weapons. To ensure that the individual, unprotected soldier can still be equipped with such projectiles, there are so-called portable recoilless anti-tank weapons, in which the muzzle momentum is compensated by an equally large one opposite the shooting direction. Technically, this is achieved with weapons simultaneously firing the grenade and ejecting a similarly heavy mass at a similar speed opposite the direction of fire (principle of the so-called Davis gun). To accelerate a projectile without recoil, the rocket principle can also be used, in which a gas jet streams out to the rear at very high speed, thus canceling the recoil momentum acting on the shooter.

In both cases, behind the weapon, i.e., behind the shooter, arises an area of deadly danger, in which it is forbidden to stay while firing. This area depends on the weapon system and can be several 10 m in length.

In the following, two fatal accidents are reported in which a person was behind such a recoilless weapon when firing. One case with a Davis gun occurred during a Bundeswehr practice shooting, and the other was caused by a rocket tube during a Swiss Army shooting exercise.

## Case reports

### Case 1

The training area Wildflecken is a more than 7000-ha large military practice area in Northern Bavaria. In mid-May 2017, a multi-day combat exercise took place on the training area instead, in which, inter alia, a 22-year-old soldier with the military rank of a so-called Hauptgefreiter participated. The combat exercise, which included the launching of Panzerfaust PzF 3, a Davis gun from Dynamit Nobel Defence (DND), had been carried out by all practitioners—both the soldiers and the trainers involved—several times; all participants in the exercise were considered to be experienced in this specific exercise.

The later injured soldier was about to launch a Panzerfaust PzF 3 right next to a comrade in a row. First, the later casualty fired his Davis gun without complications. Immediately thereafter, his comrade on the left next to him began to prepare himself for the launch, when the soldier, for reasons unknown and unanswered, left his post and stepped behind his firing comrade. Those soldiers, who were in the wider area of the rear of the soldier being just about to shoot, called to the comrade immediately before the shot “Stopfen, Stopfen,” which meant that he should stop the shooting process, for which it had been too late however. The 22-year-old Hauptgefreite, who had reportedly been about 1.5 m behind the Davis gun at the time of firing, was hit by the counter-mass of them and thrown back about 8 m. An immediately summoned doctor of the Federal Armed Forces was only able to certify the soldier as dead on site.

#### Autopsy findings

**Fig. 1:**
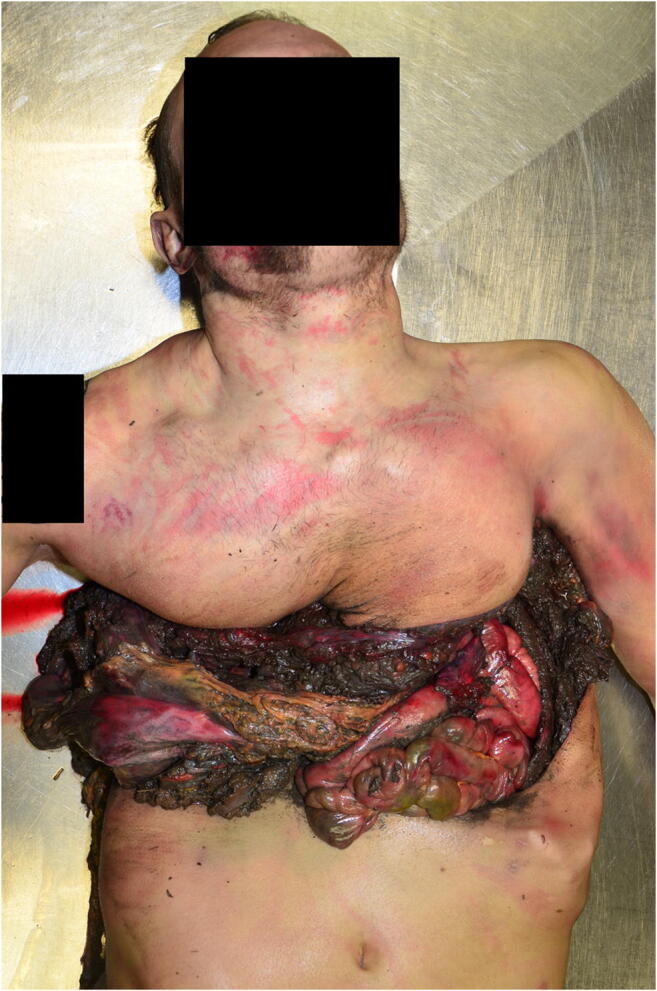
Case 1: Injuries at diaphragmatic level at the front of the body

**Fig. 2:**
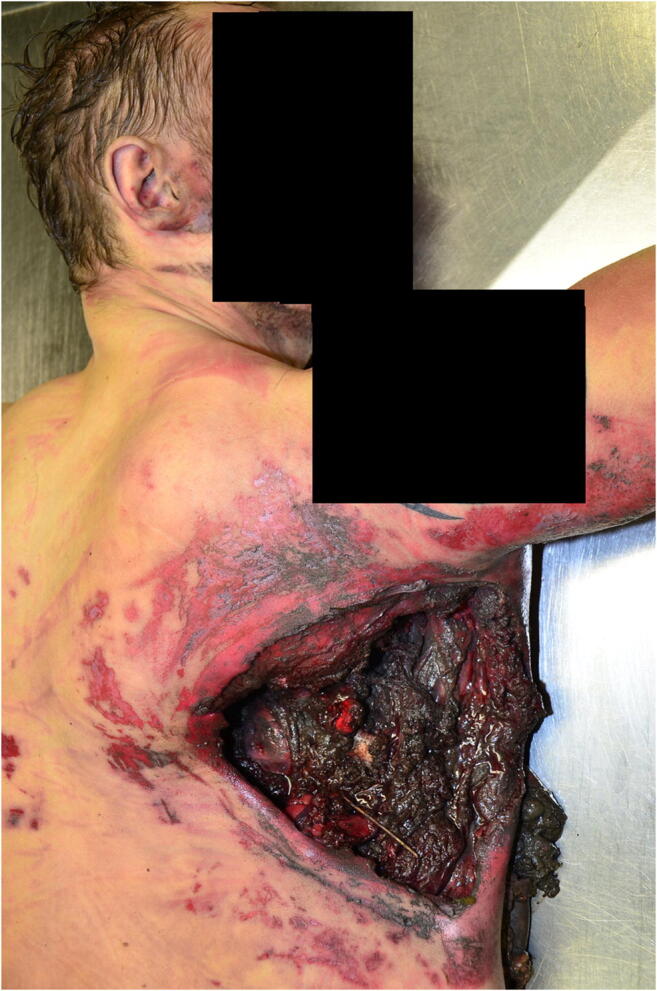
Case 1: Injury on the back of the right side of the body; reaching up to the right arm

The corpse was fully clothed with an in-the-trunk-area blood-soaked military combat outfit. On the back, in projection on the upper right back and the shoulder blade region, were extensive textile lacerations. After undressing, a large-scale destruction of the thorax and upper abdomen was to see: The abdominal organs were exposed and completely covered with a thick-layered, metallic gray material. A loop of dense olive tissue was in the depth of the abdominal wound. The ribcage was badly deformed. In the anterior thorax and abdominal area, approximately at the level of the diaphragm, a 13-cm high, transversely extending, band-like zone with deep rupture of the soft tissue delimited by a zone of blistering of the skin could be seen (Fig. 1); there was a large, almost triangular shaped, deep-reaching substance defect of 18 cm height and 20 cm width with marginal, large grayish deposits of a metallic-like material on the right lateral posterior thoracic region below the right axilla (Fig. 2). In the marginal zone of the defect, a 9-cm-wide dry and dark red zone was visible at the top, which was irregularly bounded and contained small areas of uninjured skin. Additionally, there was a dry dark red zone of the injured skin at a width of 8 cm down to the right humerus.

**Fig. 3:**
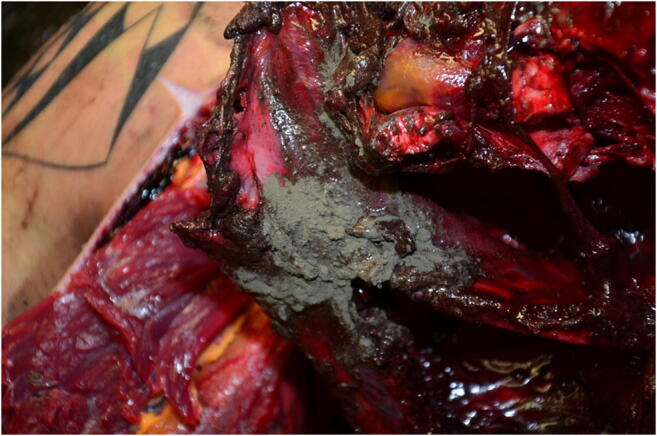
Case 1: Deposits of thick, gray, powdery material, partially compressed in the wound

In the thoracic cavity were found deposits of a gray, metallic, powdery material consisting of bright plastic spheres and textile ribbons pressed together to form slabs (Fig. 3). The heart had been torn open on its left side, the walls of the atrium and the atrium itself were nearly completely destroyed, and the first part of the large vessels and coronary arteries showed several transversal fissures. The aorta in the chest region and the lungs showed large-scale tissue destructions on both sides; only parts of residual tissue with separated main bronchi could be identified. In the large wound of the upper abdomen and the lower chest region, the intestines were largely torn out from their anatomical relation. The liver was present in parts, which were covered with a blackish-grayish, metallic shiny layer. The stomach was torn open; the tissue coated from the outside with a grayish material. Pancreas and adrenals were no longer identifiable. The aorta in the upper abdominal area was also torn. Both kidneys showed tissue destruction at the upper pole. The spine was broken between the 6th and 12th thoracic vertebra, the bony intermediate part of the spine was block-like detached. Both clavicles were broken, all ribs were broken in several pieces, and the chest bone was smashed.

According to police investigations, no evidence of a suicidal motive of the deceased could be found. His social environment neither had been instable; nor was he described as reckless or psychologically conspicuous in the troupe. A forensic-toxicological analysis of body fluids of the deceased did not detect any drugs.

### Case 2

**Fig. 4:**
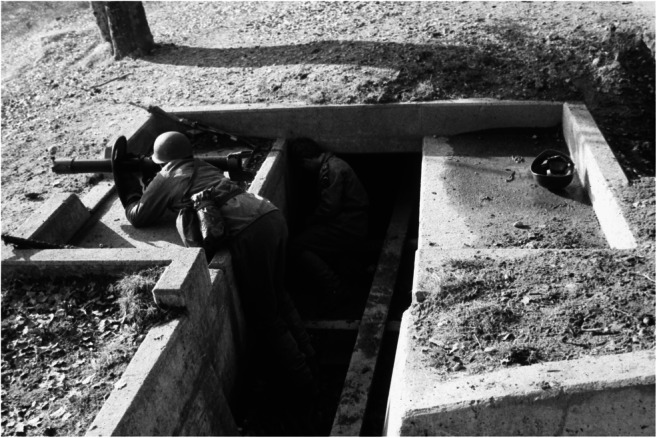
Positions of the shooter and loader during a firing exercise with an 83-mm rocket tube 80

In the morning at 26 of February 1991, an accident occurred at the “Sand”, a Swiss army training area and shooting place near Bern in which a 24-year-old corporal was seriously injured and died. The Swiss army portable anti-tank weapon at that time was an 83-mm rocket tube 80 that was operated by a shooter and a loader. To shoot an anti-tank rocket, the loader had to load the rocket from behind into the tube and then to give a slap to the shooter as a sign for ready to shoot. Then, he had to stay beside the weapon until the rocket was shot (Fig. 4). During an exercise within fixed prepared shooting positions in trenches, the corporal, who later died, operated as loader, did all manipulations correctly including the sign for ready to shoot. Then, suddenly he stood up and went for unknown reasons behind the rocket tube exactly at the moment when the shooter ignited the rocket.

He was hit by the propulsion jet of the rocket and hurled against the back wall of a trench, where he remained lying down seriously injured. The helmet was torn away through the blast. Immediately after the accident, the severely injured corporal had no pulse. While doing first-aid measures, the helpers noticed that liquid blood was escaping from the mouth of the corporal during ventilation. In consequence, artificial respiration was stopped. When the corporal arrived at the emergency ward at the hospital 30 min later, he was certified as dead.

#### Autopsy findings

**Fig. 5:**
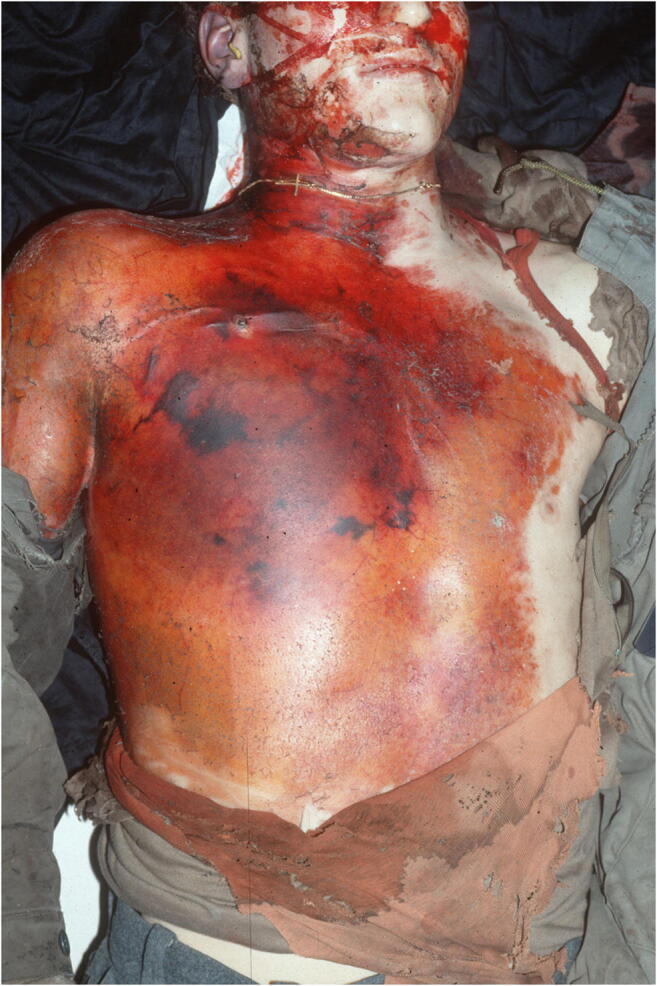
Case 2: Third degree burns resulting from the jet blast

**Fig. 6:**
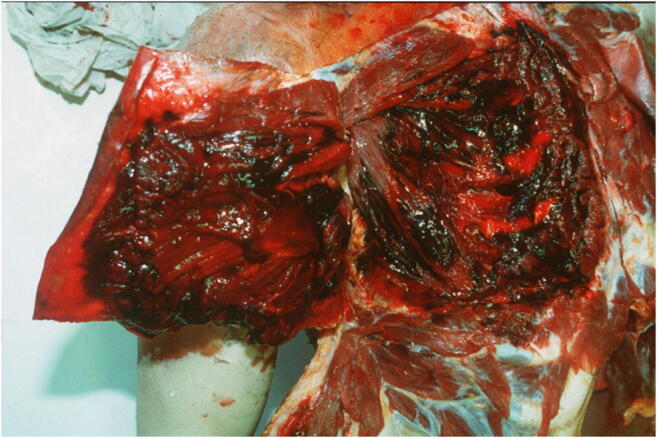
Case 2: Bleedings in the pectoral muscles

The main findings of the autopsy were burns of the skin in the area of the face and the right chest as well as a channel-like defect of the skin, chest muscles, intercostal space, chest organs, and vessels up to the bifurcation of the thigh veins in the pelvic area with massive blood accumulation in the chest (Figs. 5 and 6). Except a small metal crescent at the skin defect of the right chest, no other macroscopic or radiological detectable metal components or projectiles could be found. The small metal crescent was interpreted as a part of the ignition cartouche that was thrown backwards after the shot.

The injuries were seen as consequences of pressure development or of the extremely fast gas jet; therefore, a technical examination was carried out: On the occasion of a series of tests carried out by the Ballistic Lab of the Ministry of Defence in Thun, Switzerland, a massive pressure effect in the area of the propulsion jet of the rocket behind the weapon could be shown. The results of the forensic medicine could be easily reconciled with such a pressure effect. It was quite conceivable that the channel-shaped defect could have even occurred without a projectile as the area was small and the pressure was high. It was remarkable that the canal was not straight. Instead, the gas jet sought the path of lowest (tissue) resistance and led to bleeding, especially at the vessels, at suspension points or fixed anatomical sites. As a result of the injuries, especially in the area of the right lung and the heart, massive blood accumulation occurred in the chest area and led ultimately to internal bleeding. The descriptions from the comrades who administered first aid that blood was leaking from the mouth of the injured corporal during artificial respiration are compatible with the accumulation of blood in the chest area with partial opening of the trachea branches. The extent of the injuries was so severe that death occurred immediately after the accident. Despite adequate immediate life-saving procedures, no rescue would have been possible. No pathological findings of the inner organs could neither macroscopically nor microscopically be detected, resulting in the opinion that death was the exclusive and immediate consequence of the accident. Due to the course of the channel-like tissue defect, it can be assumed that the corporal was hit by the propulsion jet of the rocket from right to left and from top (cranial) to bottom (caudal). This injury morphology could be explained by standing up in the trench behind the weapon. The slight grazes on the occiput and the back of the trunk were compatible with the secondary impact on the back wall of the trench. The band-shaped superficial skin abrasion on the chin could be explained by the chin band of the helmet and spoke for its correct wearing. Blood alcohol determination showed a negative result and in the chemical toxicological examinations, no drugs could be detected.

In summary, it can be stated that due to forensic medical findings, the corporal was caught by the propulsion jet of the rocket and injured by the massive pressure effect. The pressure caused a channel-like defect in the chest wall, chest area, and abdominal area with massive injury to the lungs and heart, among others.

## Physical data of the involved weapons

### General

Recoilless portable anti-tank weapons are usually equipped with 1.5–4.5-kg grenades, which are fired at muzzle velocities in the range of 100–200 m/s. This results in muzzle energies of about 30–55 kJ and the muzzle momentum which is decisive for the recoil of about 300–650 Ns (for comparison: the muzzle energy of a rifle is approx. 1.5–3.5 kJ, the muzzle impulse 3.5–7.5 Ns).

### The Davis gun “Panzerfaust 3” (PzF 3)

The Panzerfaust PzF 3 of Dynamit Nobel Defence (DND), which was used in the first accident case presented here, is a recoilless, shoulder-mounted anti-tank weapon, which can be carried by a soldier and be fired from within closed rooms [1]. The caliber of the tube is 60 mm the one of the grenade 110 mm. The weight is 12.9 kg. The combat distance of the Panzerfaust PzF 3 is up to 400 m according to the manufacturer.^1^ The total mass of the Panzerfaust which is relatively large in comparison with the payload and the two opposite acceleration sections lead to relatively long tubes. A safety distance to the weapon must be held [1]. Due to a novel damming, the Panzerfaust PzF 3 can also be used in closed rooms.

**Fig. 7:**
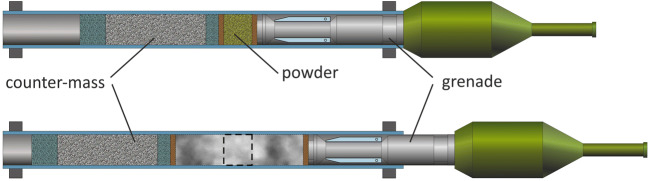
Construction of a Davis gun (schematic)

The ammunition of the Panzerfaust consists of the projectile, the propellant charge, and a so-called inert counter-mass. The burnup of the propellant charge between the projectile and the counter-mass leads to a pressure which accelerates oppositely projectile and counter-mass (Fig. 7). The Panzerfaust can be adapted to a variable recoil compensation (time, distance, force) by choosing adjustable parameters masses, speed, and distances of the counter-mass as well as the projectile way.

In order to compensate the recoil, a momentum corresponding to the muzzle momentum in the opposite direction must be generated at the time the shot is fired. For the PzF 3 (a Davis gun) involved in the first case presented, the mass of the standard grenade is 3.8 kg; it is fired with a muzzle velocity of 165 m/s. The muzzle momentum is therefore 627 Ns, and the muzzle energy 51.7 kJ. The counter-mass consists of approx. 3 kg iron powder in a fabric bag. There is no recoil if the counter mass is ejected at about 210 m/s. Their energy thus amounts to about 65.5 kJ.

One can roughly assume that already a few meters behind the tube the energy of the counter mass has halved (reduction of the effective mass due to spread out, reduction of the speed) and still amounts to about 30–35 kJ.

### The 83-mm rocket tube 80

**Fig. 8:**
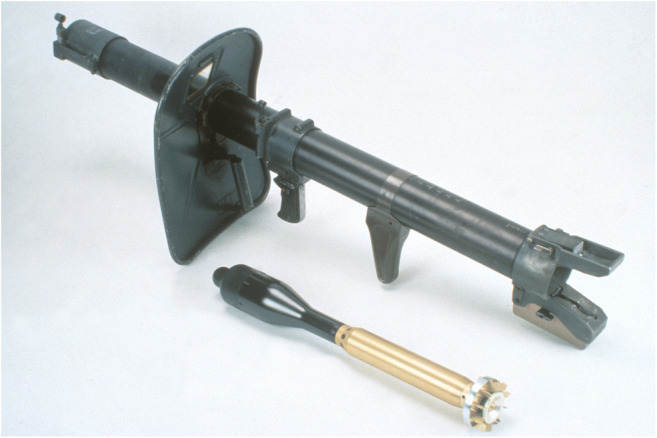
The Swiss Army 83-mm rocket tube 80

The second case discussed in this publication occurred when shooting with a rocket tube (Fig. 8). The rocket leaves the muzzle with a velocity of 110 m/s and a mass of 1.74 kg. At this moment, the momentum is 191 Ns and the energy 10.5 kJ. The propulsion jet leaves the nozzle at a velocity of approx. 1900 m/s in the opposite direction to the shot; this leads to a backward energy flux of approx. 9 kJ/ms. During the first 5 ms, the rocket moves 6 cm ejecting a gas jet of 45 kJ energy to the rear, which has a very high pressure and a temperature of more than 1000 °C. The energy decreases rapidly, but will still be in the order of 20–30 kJ within the first 50 cm.

In both cases, the victims would be exposed to energies around 30,000 J, in the first case by kinetic energy and in the second by pressure energy of the high-velocity jet associated with a high temperature.

## Discussion

Injuries described in the presented accidents are rare among today’s European legal-medicine autopsies. Due to the great force of the recoil of the Panzerfaust and the too small safety distance of the soldier to the weapon in the first case, the internal organs were almost completely destroyed. Since injuries by weapons with this destructive potential are usually found in war zones, they are not often described in detail morphologically. Literature review revealed how rare is scientific documentation of fatal injuries caused by portable recoilless anti-tank weapons [2–4].

In 2007, a comparable casuistry to the second case presented was published: A 26-year-old man suffered from injury by a propulsion jet of a rocket on his right thigh due to an RPG-7. The patient had already suffered “significant blood loss” in the field and tried to control the bleeding with a tourniquet but was unsuccessful. Upon arrival of the care team, the patient had a Glasgow Coma score of 14, a systolic blood pressure of 80 mmHg, and a hematocrit of 30%. Physical examination revealed a severely contaminated circumscribed soft tissue injury of the femur with active hemorrhage. The patient became unconscious and had to be resuscitated due to an onset of hemorrhagic shock. An isolated bony injury revealed a splintered femoral fracture in the middle of the shaft. The distal end of the injury was insensitive and without pulse. During bleeding control and debridement in the operating room, it was found that the limb was held only by a superficial skin bridge and a narrow subcutaneous soft tissue bridge and was thus subtotally amputated [4]. In the case presented in this paper, extensive soft tissue and bony injuries were found as well. In literature, however, so far, no case was described in which also shiny metallic material of the counter-mass of the projectile was found in the depth of the wound.

For reconstruction of accidents like described above with determination of inter alia position of shooter and shooting angle, the representation of rejects, firing channel, firing direction, and firing distance is of great importance [5, 6]. In the autopsy of the victim of the first case presented, the right posterior thorax side could be identified as entry region with a pronounced drying zone extending to the right upper arm and an almost triangular substance defect; the exit region was on the front of the body. Thus, the position of the injured person could be reconstructed with his back to the Panzerfaust, which can support the thesis of an accidental event. Firing distance can be plausibly reconciled with the information on the course of events (distance at firing approx. 1.5 m to the weapon); the recoil angle is to be regarded as slightly decreasing, which corresponds to the usual position of the Panzerfaust at firing with the barrel raised forward and thus decreasing recoil angle. Because in the second case the shooting of the rocket happened in a trench, the loader stud quite close behind the rocket tube at the moment of the shot. He was hit in a distance of approximately 0.5–0.8 m.

Ultimately, it can be stated that in both cases presented, two different causes of death were present: massive blunt violence in the first case versus a hot gas jet of very high pressure and speed in the second case.

## Conclusion

The two accidents presented here illustrate that a comprehensive evaluation of this special and seldom type of injury can only lead to reliable results within an interdisciplinary approach consisting of investigating authorities, military authorities, ballistics, and forensic medicine.

There was no description of the metallic material found in the wound in the first case presented so far, which can be assigned to the counter-mass of the Davis gun. The extraordinary wound morphology in the presented cases is to see as relevant not only to forensic medicine but also to emergency surgery.

The tragedy of both described accidents clarifies besides the devastating mortal potential behind portable recoilless weapons the importance of observance of safety regulations during combat exercises.

## Footnotes

1 http://dn-defence.com/pzf-3-96.html; November 2019
